# A Novel CXCL10-Based GPI-Anchored Fusion Protein as Adjuvant in NK-Based Tumor Therapy

**DOI:** 10.1371/journal.pone.0072749

**Published:** 2013-08-30

**Authors:** Niklas Muenchmeier, Sophia Boecker, Lorenz Bankel, Laura Hinz, Nicole Rieth, Constantin Lapa, Anna N. Mendler, Elfriede Noessner, Ralph Mocikat, Peter J. Nelson

**Affiliations:** 1 AG Klinische Biochemie, Medizinische Klinik und Poliklinik IV, Klinikum der Universität München, Munich, Germany; 2 Institut für Molekulare Immunologie, Helmholtz-Zentrum München, Deutsches Forschungszentrum für Gesundheit und Umwelt, Munich, Germany; University of Leuven, Rega Institute, Belgium

## Abstract

**Background:**

Cellular therapy is a promising therapeutic strategy for malignant diseases. The efficacy of this therapy can be limited by poor infiltration of the tumor by immune effector cells. In particular, NK cell infiltration is often reduced relative to T cells. A novel class of fusion proteins was designed to enhance the recruitment of specific leukocyte subsets based on their expression of a given chemokine receptor. The proteins are composed of an N-terminal chemokine head, the mucin domain taken from the membrane-anchored chemokine CX3CL1, and a C-terminal glycosylphosphatidylinositol (GPI) membrane anchor replacing the normal transmembrane domain allowing integration of the proteins into cell membranes when injected into a solid tumor. The mucin domain in conjunction with the chemokine head acts to specifically recruit leukocytes expressing the corresponding chemokine receptor.

**Methodology/Principal Findings:**

A fusion protein comprising a CXCL10 chemokine head (CXCL10-mucin-GPI) was used for proof of concept for this approach and expressed constitutively in Chinese Hamster Ovary cells. FPLC was used to purify proteins. The recombinant proteins efficiently integrated into cell membranes in a process dependent upon the GPI anchor and were able to activate the CXCR3 receptor on lymphocytes. Endothelial cells incubated with CXCL10-mucin-GPI efficiently recruited NK cells *in vitro* under conditions of physiologic flow, which was shown to be dependent on the presence of the mucin domain. Experiments conducted *in vivo* using established tumors in mice suggested a positive effect of CXCL10-mucin-GPI on the recruitment of NK cells.

**Conclusions:**

The results suggest enhanced recruitment of NK cells by CXCL10-mucin-GPI. This class of fusion proteins represents a novel adjuvant in cellular immunotherapy. The underlying concept of a chemokine head fused to the mucin domain and a GPI anchor signal sequence may be expanded into a broader family of reagents that will allow targeted recruitment of cells in various settings.

## Introduction

Cell-based immunotherapy harnesses the natural cytotoxic potential of immune cells to eliminate target cells in a highly specific manner. In addition to T lymphocytes, the activity of NK cells is desirable as they play a complementary role to CTL in the antitumor response by recognizing tumors which are resistant to T cell killing due to downregulation of MHC class I molecules [1]–[3]. A problem frequently encountered using cytotoxic lymphocytes as anti-tumor agents is insufficient infiltration of the tumor tissue, in particular evident for NK cells [4]–[8], which has been proposed as an explanation for the lack of efficacy of cellular tumor-therapy in many settings [9]–[11]. This has been linked to changes in the tumor vasculature leading to reduced expression of adhesion molecules on tumor endothelial cells, as well as reduced efficacy of proinflammatory cytokines in upregulating adhesion molecule expression [6], [12]–[15].

**Figure 1:**
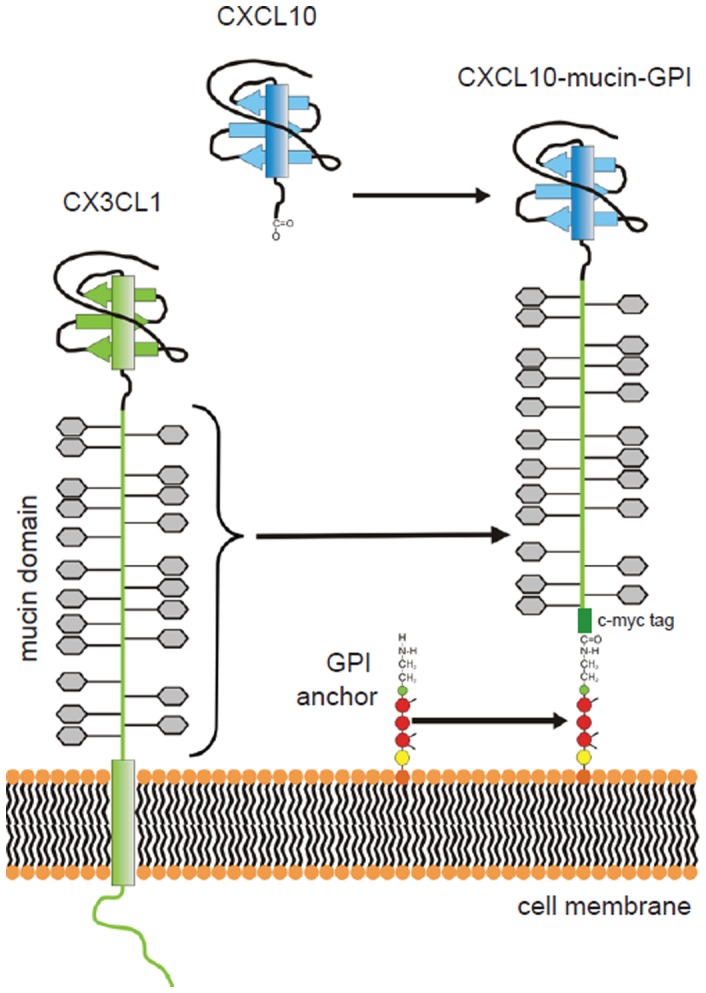
Composition of CXCL10-mucin-GPI as an example for a novel class of GPI-anchored chemokine fusion proteins. The mucin domain of CX3CL1 was combined with a GPI anchor and a CXCL10 chemokine head to generate a flexible tool for the modification of tumor micromilieus capable of selectively stimulating the recruitment of CXCR3^+^ leukocytes. The chemokine head directs the specificity towards CXCR3^+^ leukocytes, while the mucin domain assists in the recruitment process and lowers the requirement for other adhesion molecules. Inclusion of a GPI anchor allows the protein to integrate into the cell membranes of tumor, stromal and endothelial cells when applied exogenously, thus superseding the transfer of genetic material into the tumor.

We describe here an example of a novel class of reagents designed to selectively recruit leukocytes based on chemokine receptor expression (Figure 1). We use fusion proteins whose backbone is a mucin domain derived from the chemokine CX3CL1 (Fractalkine), which has the ability to capture and recruit CX3CR1^+^ leukocytes under physiological conditions with reduced requirement for additional adhesion molecules such as ICAM-1 or VCAM-1 [16]–[18]. It has been shown that the specificity of that protein can be redirected from CX3CR1^+^ leukocytes to leukocytes expressing other chemokine receptors by exchanging the N-terminal chemokine domain for an unrelated chemokine [16]. In the current study we fused a CXCL10 chemokine head to the mucin-like stalk of CX3CL1, thereby redirecting the recruitment tropism of the molecule towards leukocytes expressing the CXCL10-specific receptor CXCR3 (Figure 1). Furthermore, the transmembrane-domain of CX3CL1 was exchanged for a C-terminal glycosylphosphatidylinositol (GPI) anchor signal sequence. Purified GPI-anchored proteins possess the ability to integrate spontaneously into the cell membranes of virtually any cell eliminating the need for transfection [19].

Following expression and purification, the recombinant CXCL10-mucin-GPI protein readily incorporated into cell membranes and effectively fostered the direct recruitment of CXCR3^+^ NK cells.

## Materials and Methods

### Ethics Statement

The research meets all applicable standards for the ethics of experimentation and research integrity. The animal studies were approved by and conducted in accordance with the principles of the regulatory agency of the State of Bavaria, Germany. The human T cell lines JB4 [20] and DS4 were derived from healthy donors after written informed consent was obtained with respect to taking the samples and making the cell line and upon approval by the Ethics Committee of Ludwigs-Maximilians-University, Munich.

### Molecular Cloning Strategies

Recombinant proteins were generated in Chinese Hamster Ovary (CHO) cells. The PCR-based cloning techniques and selection criteria used to generate the series of fusion-protein constructs are described in Materials and Methods S1.

### CXCR3 Receptor Internalization Assays

Coincubations of human CXCR3^+^ CD8^+^ CTLs (JB4, previously published in [20]) with non-transfected CHO cells or CHO cells stably transfected with CXCL10-GPI or CXCL10-mucin-GPI were performed as readout for bioactivity of the chemokine domain. 6×10^5^ CHO cells in 25 µl RPMI 1640 (Invitrogen, Carlsbad) were transferred into 96-well round bottom plates. 25 µl recombinant human CXCL10 (Peprotech, Hamburg, Germany) at concentrations of 1.5 µg/ml or 200 ng/ml in RPMI 1640 served as positive control, and 25 µl RPMI 1640 as negative control. All samples were prepared in duplicates. 2×10^5^ JB4 cells in 25 µl RPMI 1640 were added to each well prepared as described above. The plate was incubated at 37°C and 5% CO_2_ for 30 min. All subsequent steps were performed on ice. The cells were washed and resuspended in FACS buffer (2 mM EDTA, 2% FCS in PBS). 7-AAD and FITC-labeled CD3-specific antibodies (BD Pharmingen, Bedford, USA) were then added to all samples. Among each pair of duplicates, one sample additionally received RPE-labeled, CXCR3-specific antibody (clone 1C6, BD Pharmingeņ Bedford. Additional control experiments presented in Materials and Methods S1 used clone 49801; R&D Systems, Minneapolis), while the other received an RPE-labeled isotype control (BD Pharmingen, Bedford, USA). FACS analysis was then used to identify (CD3-positive) JB4 cells and the mean fluorescence intensity (MFI) of CXCR3 on these cells was determined. Internalization was expressed as percent reduction of CXCR3 MFI compared to the medium/non-transfected controls.

### Protein purification

The GPI-anchored fusion proteins were purified using Fast Protein Liquid Chromatography (FPLC). This was facilitated by the inclusion of a double c-myc epitope tag integrated into all constructs, and the use of anti-c-myc affinity chromatography. The specific methods are detailed in Materials and Methods S1.

### Incorporation of purified proteins Into Cell Membranes

FACS staining was used to assess the ability of purified recombinant fusion proteins to incorporate into cell membranes. CHO or endothelial cells were detached and resuspended in prewarmed serum-free culture medium. 1×10^6^ CHO cells or 5×10^5^ endothelial cells per well were transferred into a sterile 96 well round bottom plate and the cells were resuspended in 100 µl of serum-free medium containing defined concentrations of purified recombinant fusion proteins or a buffer control. All samples contained the same percentage of chromatography buffer. The cells were incubated at 37°C and 5% CO_2_ for 1–1.5 h, washed and resuspended in FACS buffer containing monoclonal antibodies (anti c-myc: clone 9E10, purified in house), or isotype-matched control antibodies (Sigma-Aldrich, Taufkirchen, Germany). Following incubation for 45 min at 4°C, the cells were washed and resuspended in FACS buffer containing RPE-labeled secondary antibodies (Dako, Roskilde, Denmark, 10 µg/ml) and 7-AAD. After 30 min incubation at 4°C, the cells were washed again and analyzed.

Immunofluorescence microscopy was used to detect the subcellular localization of the incorporated proteins. Primary microvascular endothelial cells were grown in culture dishes with high fluorescence permeability (Ibidi, Martinsried, Germany). Subsequently, the cells were washed and incubated for 1.5 h with purified CXCL10-GPI (1.8 nM), CXCL10-mucin-GPI (0.8 nM) or a buffer control, all diluted in culture medium and all containing the same percentage of buffer to exclude artifacts. Following treatment, the cells were washed and fixed with 1% Paraformaldehyde. Incorporated proteins were then detected using anti c-myc primary and biotinylated secondary antibodies in combination with RPE-labeled streptavidin.

### Laminar flow Assays

Laminar flow assays were performed in analogy to previously described experiments [21]–[25]. Experiments were performed using either primary microvascular endothelial cells from fetal foreskin, selected for blood vessel endothelial cells (CD31^+^, von-Willebrand factor^+^, Podoplanin^-^, Smooth muscle actin^-^; Promocell, Heidelberg, Germany) or CHO cells (ATCC, USA).

For experiments involving endothelial cells, the cells were grown to 80% confluence in tissue culture flasks. 24 h prior to the experiment, the cells were seeded into microscope slides (µ-slide I 0.4 ibitreat, ibidi, Martinsried, Germany), which were precoated with 12 µg/ml Collagen G (Biochrom, Berlin, Germany) for 30 min. Cells were split at an area ratio of 1:0.8 into the slides. 1 h prior to the analysis, the slides were treated with purified GPI-anchored proteins diluted in serum-free culture medium.

For experiments assessing adhesion to transfected CHO cells, the cells were grown to 90% confluence. One day before the assay, the cells were seeded at a ratio of 1:1 into the slides.

HBSS buffer (Sigma, Taufkirchen, Germany) supplemented with 50 mM HEPES (Invitrogen, Carlsbad, CA, USA) and 0.5% BSA (Invitrogen, Carlsbad, USA) was used for flow studies. CXCR3^+^ CD8^+^ CTLs [DS4, unpublished], were resuspended in assay buffer to yield a concentration of 2×10^5^. This cell line was derived from mixed lymphocyte/tumor cells cultures using PBL of a healthy donor and the allogeneic human renal cell carcinoma cell line RCC26. Following the initial priming, CD8+ T cell clones were established by limiting dilution cloning and are routinely cultured in the laboratory of Dr. Noessner. We are happy to provide this line upon request. The NK cell line YT (ATCC 434; DSMZ GmbH, Braunschweig, Germany), or primary murine NK cells (freshly isolated from the spleens of C57BL/6 wt mice using magnetic beads) were used in concentrations of 2.6×10^5^ or 3x10^5^ cells/ml, respectively. The cells were kept in a water bath at 37°C throughout the experiment. Experiments were performed at 37°C in an incubation chamber (ibidi, Martinsried, Germany) mounted on the stage of an inverted microscope equipped with a digital video camera (Horn Imaging, Aalen, Germany). Each slide was first flushed with assay buffer using a programmable syringe pump (WPI, Sarasota, USA) to remove cell debris. Subsequently, leukocyte suspension was flushed into the slide. The flow rate was then lowered to yield the desired shear stress (indicated in each figure) and the interactions were recorded for 5 min.

Videos were used to determine the number of leukocytes accumulating in the respective field of view. Tight adhesion was defined as an event in which a particular NK cell adhered to the endothelium and did not move further than one cell diameter within 30 sec. Rolling adhesion was defined as an event in which the NK cell adhered to the endothelium, but was dragged along the endothelium by the shear forces exerted by the buffer at a higher speed than one cell diameter per 30 sec or detached again. Cells displaying both rolling and tight adhesion were counted only as tightly adherent.

### *In vivo* Analysis

Murine 291 B cell lymphoma cells [26] were implanted in female wt C57BL/6 mice (Taconic, Ry, Denmark) between 11 and 21 weeks of age. 10^7^ 291 cells in 150 µl PBS were injected subcutaneously into each flank of the mice. Tumor growth was monitored by palpation and the experiments were initiated once the tumors had reached a diameter of approximately 8 mm. Purified CXCL10-mucin-GPI (0.23 pmol) in 50 µl PBS supplemented with 0.025% Triton X-100h was injected into the center of each tumor. As controls, either the same or a 500×higher molar quantity (115 pmol) of commercially available human CXCL10 (rhCXCL10) were injected. Additional controls included completely untreated tumors and injection of the same volume of identically purified sEGFP-GPI. Three separate tumors were treated with each protein or control. After 4 h, the mice were sacrificed and the tumors were removed.

### FACS Analyses of Tumors

For FACS analysis, the tumors were minced, filtered through a 40 µm cell strainer (Becton Dickinson, Heidelberg, Germany), washed in PBS (Life Technologies, Darmstadt, Germany), depleted of red blood cells and washed in PBS again. Cells were stained with ethidium monoazide for dead cell exclusion prior to labeling with specific antibodies for CD3 (eF450), CD8 (AF488) (both from eBioscience, Frankfurt, Germany), CD4 (PerCP), NK1.1 (PE) (both from Becton Dickinson, Heidelberg, Germany) and CXCR3 (APC) (BioLegend, Fell, Germany). Staining was performed in FACS buffer consisting of PBS, 2% FCS, 0.1% sodium azide and 5 mM EDTA. FACS analyses were performed using a LSR II (Becton Dickinson, Heidelberg, Germany) and data were analyzed using FloJo 8.8 software (TreeStar, OR, USA).

### Histology of Tumor Sections

For histology, the tumors were fixed for 24 h in 10% neutral buffered formalin. The presence of NK cells and T cells was detected using immunohistology with antibodies specific for NKp46 and CD3, respectively. The methods detailing the various procedures and reagents used are available in Materials and Methods S1.

## Results

### Expression of the Fusion Constructs in CHO Cells

**Figure 2:**
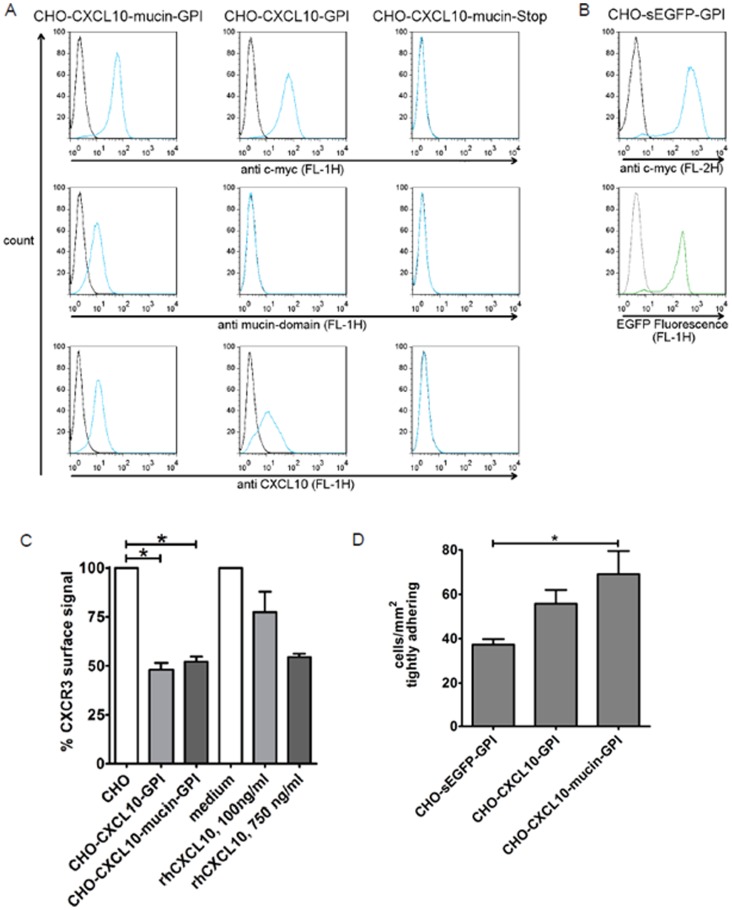
The recombinant GPI-anchored proteins are present on transfected CHO cells and activate CXCR3. **A**: Stably transfected CHO cells expressing the GPI-anchored CXCL10 constructs (CXCL10-GPI or CXCL10-mucin-GPI) or a control lacking the GPI anchor (CXCL10-mucin-Stop) were incubated with antibodies against the c-myc epitope tag, the mucin domain, the CXCL10 chemokine head or matching isotype controls. Bound antibodies were detected by staining with FITC-conjugated secondary antibodies and the fluorescence intensity was measured by FACS. Black lines indicate staining with isotype controls, blue lines indicate staining with specific antibodies. **B**: In the case of CHO cells transfected with the GPI-anchored version of eGFP (sEGFP-GPI), RPE-conjugated secondary antibodies were used to detect the c-myc epitope tag (anti c-myc antibodies: blue line, isotype control: black line) while the fluorescence by the EGFP-protein was measured in the FL-1 channel (green line) and compared to non-transfected CHO cells (grey line). All histograms are gated on viable cells identified by 7-AAD exclusion. **C**: CXCR3 on human T cells is internalized after coincubation with cells expressing CXCL10-GPI or CXCL10-mucin-GPI. Human CXCR3^+^ CTL (JB4) were incubated with CHO cells stably transfected with the GPI-anchored CXCL10 fusion constructs (CHO-CXCL10-GPI or CHO-CXCL10-mucin-GPI) or non-transfected CHO cells for 30 min. Other samples were incubated with commercially available soluble CXCL10 (rhCXCL10) at the indicated concentrations. The CXCR3 signal on the T cells was subsequently determined by FACS and related to the CXCR3 signal on T cells incubated with non-transfected CHO cells or medium without chemokine, respectively. Bars represent averages of two (CHO-CXCL10-GPI and 100 ng/ml rhCXCL10) or three (all other conditions) independent experiments +/− standard deviations. Statistical significance was calculated using the Kruskal-Wallis test (P = 0.0167), followed by Dunńs post test; * = P<0.05. **D**: Adhesion of CXCR3^+^ T cells (DS4) is stimulated by contact with the GPI-anchored CXCL10 fusion proteins. CHO cells transfected with CXCL10-GPI, CXCL10-mucin-GPI or sEGFP-GPI as control were grown to confluence in channels with defined geometry. T cells were drawn into the channel, left to adhere for 5 min under static conditions and subsequently subjected to a shear stress of 1 dyn/cm^2^. T cells that were still adherent after 2 min of flow were counted and the results were expressed as cells/mm^2^. The bars represent averages from three independent experiments +/− standard deviations. Statistical significance was calculated using the Kruskal-Wallis-test (P = 0.039) followed by Dunńs post test; * = P<0.05.

DNA constructs of CXCL10-mucin-GPI and controls in which the mucin domain or the GPI anchor signal sequence was deleted (CXCL10-GPI and CXCL10-mucin-Stop, respectively) or in which the GPI anchor was fused to an eGFP protein (sEGFP-GPI) were generated and stably expressed in dihydrofolatereductase-deficient Chinese hamster ovary (CHO^dhfr−/−^) cells. FACS surface staining was used to identify the expressed proteins on the cell surface (Figure 2A and B). The various subunits (chemokine head, mucin-like stalk, and c-myc epitope tag) of the fusion proteins were detected using a panel of monoclonal antibodies (see Materials and Methods S1). All GPI-anchored proteins, but not the control protein CXCL10-mucin-Stop lacking a GPI anchor, were detected on the surface.

### The Engineered Fusion Proteins Induce Receptor Internalization and Adhesion

The ability of the CXCL10-based recombinant proteins to induce specific internalization of CXCR3 was used to demonstrate functionality. The experiments were performed in analogy to previously published experiments with soluble CXCL10 [27]–[30]. CXCR3^+^ human CTLs (JB4) were incubated either with stably transfected CHO cells expressing CXCL10-GPI or CXCL10-mucin-GPI or with soluble CXCL10 as positive control (Figure 2C). The CXCR3 surface signal was subsequently determined by FACS analysis using a PE-conjugated monoclonal antibody and the degree of internalization was calculated. The signal intensities after coincubation with non-transfected CHO cells or medium were set as 100%. A significant decrease in CXCR3 surface staining was seen after coincubation with CHO-CXCL10-GPI or CHO-CXCL10-mucin-GPI cells indicating bioactivity of the chemokine domain. A similar degree of internalization required 750 ng/ml (86 nM) soluble CXCL10. Similar results were obtained when using a different CXCR3-specific antibody (Figure S1). As an additional control, the assay was performed on ice, by which the loss of surface signal intensity was strongly attenuated. This indicated an active internalization process rather than a blockade of the antibody binding site by the receptor-bound chemokines (Figure S1). Moreover, we also observed a sustained calcium response in the T cells when coincubated with transfected CHO cells (Figure S2), indicating signal transduction from the activated receptor.

The ability of the recombinant GPI-anchored CXCL10 fusion protein to induce adherence to a cell monolayer was used as an additional indicator of bioactivity of the chemokine domain. Transfected CHO cells expressing CXCL10-GPI, CXCL10-mucin-GPI or sEGFP-GPI were grown to confluence in channels with defined geometry. CXCR3^+^ CTLs (DS4) were then drawn into the channel and left to adhere for 5 min under static conditions. The cells were then subjected to physiologic shear stress (1 dyn/cm^2^). The detachment of the cells was monitored by video microscopy and cells adherent after 2 min of flow were determined (Figure 2D). On average 69 cells/mm^2^ adhered in a shear-resistant manner to CHO cells expressing CXCL10-mucin-GPI. Significantly less (p<0.05 using the Kruskall-Wallis test followed by Dunńs post test) cells adhered to CHO-sEGFP-GPI cells used as negative control (on average 37 cells/mm^2^). An average of 56 cells/mm^2^ remained adherent to CHO cells expressing CXCL10-GPI (lacking the mucin domain). Recombinant CXCL10-mucin-GPI protein expressed on the CHO cells thus enabled the CXCR3^+^ CTLs to more efficiently adhere to the monolayer.

### Cell Surface Engineering Using the Engineered Fusion Proteins

**Figure 3:**
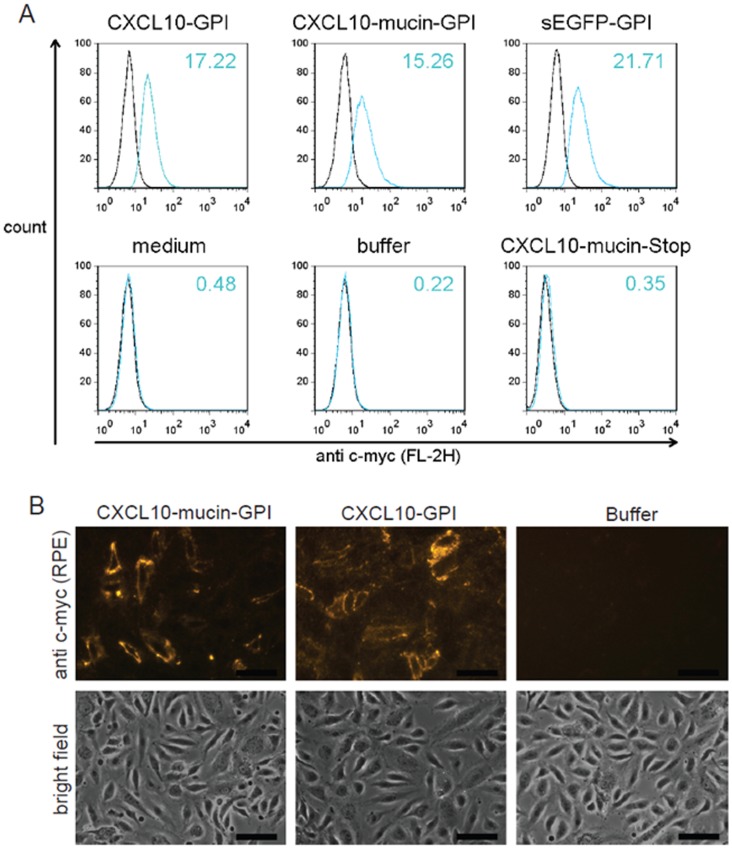
Purified GPI-anchored proteins incorporate into cell membranes. **A**: In order to test the capacity of the purified recombinant fusion proteins to reincorporate into cell membranes, non-transfected CHO cells were incubated with the purified GPI-anchored proteins (0.9 nM) for 1 h at 37°C. As controls, two samples were treated either with identically diluted chromatography buffer (buffer) or MEM alpha medium (medium). The soluble CXCL10-mucin-Stop protein served as an additional control as it lacks a GPI anchor. All samples except the medium control contained the same percentage of chromatography buffer and detergent. Following incubation, the cells were washed and tested for the presence of the proteins on their surface by FACS staining. Dead cells were identified by 7-AAD staining and the histograms shown are gated on viable cells. The black lines indicate staining with isotype-matched control antibodies, blue lines staining with anti c-myc antibodies. Mean fluorescence intensities (MFIs) are given for each sample. This experiment was performed routinely to monitor protein quality after purification and the data shown here therefore stand representative for over 20 independent experiments. **B**: To verify the subcellular localization of the incorporated proteins, immunofluorescence microscopy was performed. Primary microvascular endothelial cells were treated with purified CXCL10-GPI, CXCL10-mucin-GPI or a buffer control diluted in culture medium, with all samples containing the same percentage of buffer to exclude artifacts. After treatment, the cells were washed, fixed with Paraformaldehyde and incorporated proteins were detected using anti c-myc primary and biotinylated secondary antibodies followed by RPE-labeled streptavidin. The figure shows fluorescence images and corresponding bright field images from a representative experiment, which was performed three times. All images within each row were acquired using the same exposure time. The black bars indicate 50 µm.

Next, we determined the ability of exogenously added, purified proteins to integrate into cell membranes. Non-transfected CHO cells were incubated with purified recombinant GPI-anchored proteins. Purified CXCL10-mucin-Stop protein, which lacks the GPI anchor, was used as a control. It was applied at a higher concentration than the GPI-anchored proteins to allow potential detection of non-specific binding at low affinity. FACS staining was then used to detect the incorporated proteins on the cell surface (Figure 3A).

The CXCL10-GPI, CXCL10-mucin-GPI and sEGFP-GPI proteins were each detected on the surface of the treated CHO cells, whereas the soluble CXCL10-mucin-Stop protein was not, indicating that the membrane incorporation was mediated by the GPI anchor. Similar results were obtained using primary or immortalized microvascular endothelial cells or primary endothelial cells isolated from umbilical veins (data not shown). The staining intensities were found to correlate with the concentration of recombinant proteins used in the respective experiment (data not shown).

The subcellular localization of the incorporated proteins was then assessed by immunofluorescence microscopy. Primary microvascular human endothelial cells were used as target cells, and incorporated proteins were detected using specific antibodies (Figure 3B). The purified GPI-anchored CXCL10 fusion proteins could be detected in a membrane-associated manner on the surface of the endothelial cells, demonstrating that they had incorporated spontaneously into the cell membranes of the endothelial cells.

### Surface Engineered Endothelial Cells Recruit NK Cells Under Physiologic Flow *in vitro*

Based on our initial hypothesis, CXCL10-mucin-GPI should be able to recruit leukocytes in the absence of an inflammatory reaction, thereby overcoming endothelial cell anergy within tumor tissues. Once anchored into the cell membranes of endothelial cells, the mucin domain together with the chemokine head should concertedly induce rolling and tight adhesion of CXCR3^+^ leukocytes, followed by diapedesis into the tumor tissue. The effect of CXCL10-mucin-GPI on the recruitment of NK cells was assessed *in vitro* using laminar flow assays. The assays were conducted using resting primary human microvascular endothelial cells. These cells normally do not support the adhesion of leukocytes under conditions of physiologic flow due to the absence of selectin molecules, which are not present on resting cells [24], [25], [31]. Also in our hands, E-selectin was not detected on the resting endothelial cells by FACS analysis (data not shown).

**Figure 4:**
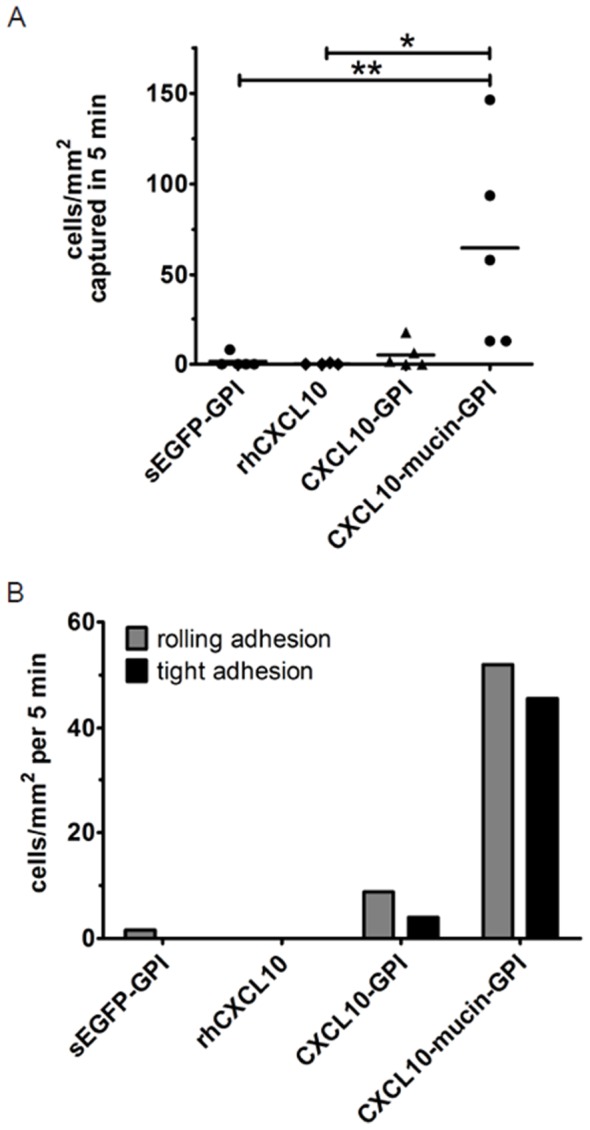
CXCL10-mucin-GPI induces rolling and tight adhesion of CXCR3^+^ NK cells under conditions of physiologic flow. Laminar flow assays were performed to test if resting primary human microvascular endothelial cells treated with the GPI-anchored CXCL10 fusion proteins could recruit freely flowing CXCR3^+^ NK cells (YT) under conditions of physiologic flow. **A**: Resting primary human endothelial cells from fetal foreskin, selected for blood vessel endothelial cells, were treated with 0.34 nM of GPI-anchored CXCL10 fusion proteins or with identically diluted sEGFP-GPI protein for 1 h. Other slides were treated with commercially available CXCL10 at 1000-fold higher concentration as additional control (rhCXCL10). All samples contained the same percentage of chromatography buffer and detergent. Subsequently, YT cells were perfused over the endothelial cells with 1 dyn/cm^2^ and the number of cells accumulating on the endothelial cells was counted. Data shown here are derived from six independent experiments, each performed using independent protein preparations and separate batches of cells. Bars represent the average numbers of cells adhering to the endothelium, +/− SEM. Statistical significance was calculated using the Kruskal-Wallis test (P = 0.0022) followed by Dunńs post test; ** = P<0.01. **B**: Experiments were performed as detailed in A. Tight adhesion was defined as an event in which a particular NK cell adhered to the endothelium and did not move further than one cell diameter within 30 sec. Rolling adhesion was defined as an event in which the NK cell adhered to the endothelium, but was dragged along the endothelium by the shear forces exerted by the buffer at a higher speed than one cell diameter per 30 sec or detached again. Cells displaying both rolling and tight adhesion were counted only as tightly adherent. Bars represent averaged values derived from four independent experiments +/− SEM. Statistical significance was calculated using the Kruskal-Wallis test (P = 0.0038 for rolling adhesion and 0.0021 for tight adhesion) followed by Dunńs post test; * = P<0.05, ** = P<0.01.

Primary human microvascular endothelial cells were incubated for 1 h with purified GPI-anchored CXCL10 fusion proteins diluted in growth medium, or with identically purified and diluted sEGFP-GPI protein. Parallel slides were incubated with commercially available recombinant CXCL10 in vehicle at a 1000 fold higher concentration, to allow detection of even minor effects mediated by conventional CXCL10. NK cells were then perfused over the endothelial cells with a shear rate of 1 dyn/cm^2^ and interactions were monitored by video microscopy (Figure 4A; readers may also refer to the exemplary video S1 which shows the adherence of YT cells to endothelial cells treated with either recombinant CXCL10 or CXCL10-mucin-GPI in real-time). On average, 60 NK cells/mm^2^ underwent adherence to endothelial cells incubated with CXCL10-mucin-GPI. By contrast, on average only 2 cells/mm^2^ adhered to the samples treated with the GPI-anchored negative control protein sEGFP-GPI. This attested that the adhesion was due to the activity of the chemokine-mucin part of the fusion protein and neither the incorporation process nor the presence of a GPI-anchored protein on the cell surface sufficiently activated the endothelial cells to induce adhesion events.

CXCL10-GPI failed to recruit significantly more cells than the two controls (4 cells/mm^2^ on average), demonstrating that the mucin domain was a prerequisite for efficient NK cell recruitment. Recombinant CXCL10 also failed to induce NK cell adherence (0 cells/mm^2^ on average), in accordance with earlier observations [23], [32]–[34].

To analyze if CXCL10-mucin-GPI was able to induce tight adhesion of captured NK cells (which is required for extravasation), the adherent cells were further characterized. Cells that displayed tight adhesion, defined as an event in which the respective NK cell did not move further than one cell diameter within 30 sec, were differentiated from those that displayed rolling adhesion, defined as an event in which the respective NK cell adhered to the endothelium, but was dragged along the endothelium by the shear forces exerted by the buffer at a higher speed than one cell diameter per 30 sec or detached again. Cells displaying both rolling and tight adhesion were counted only as tightly adherent (Figure 4B). Readers may also consult video S2 which exemplifies rolling and tight adhesion events.

Endothelial cells incubated with CXCL10-mucin-GPI induced both rolling and tight adhesion of the NK cells. On average 41 cells/mm^2^ displayed rolling adhesion, while 35 cells/mm^2^ adhered tightly to the endothelial cells. CXCL10-GPI lacking the mucin domain could only induce on average 3 events of rolling adhesion and 2 tight adhesions/mm^2^. 2 events of rolling adhesion were seen with endothelial cells incubated with the sEGFP-GPI control protein, and no adhesion at all occurred to cells treated with commercially available recombinant CXCL10. These results again indicated a dependence of the adhesion process mediated by CXCL10-mucin-GPI on the presence of the mucin domain, both for the induction of rolling as well as tight adhesion.

### The Human CXCL10-based Fusion Proteins Also Enhance the Adherence of Primary Murine NK Cells Under Flow

**Figure 5:**
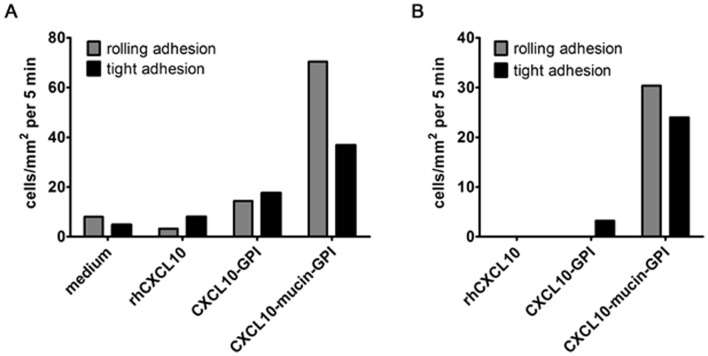
The human CXCL10 fusion proteins are bioactive in mouse. To validate that human CXCL10-based fusion proteins would be functional in mouse-based models, the adhesion of primary murine NK cells to the human-based CXCL10 fusion proteins was evaluated. Primary microvascular human endothelial cells were incubated for 1 h with 0.2 nM of CXCL10-GPI or CXCL10-mucin-GPI or commercially available human CXCL10 (rhCXCL10; 115 nM). All samples except the medium control contained the same percentage of buffer and detergent. Primary murine NK cells were then perfused over the treated endothelial cells with **A**: 0.4 or **B**: 1 dyn/cm^2^ shear stress, and adherent cells were counted. Cells with rolling adhesion were differentiated from those that adhered tightly to the endothelium as in figure 4. The figure shows the results of one of two representative experiments, with each bar summarizing data from two independent slides.

As a prerequisite for *in vivo* studies, it was then determined if the human CXCL10 fusion proteins were also active on murine cells. First, soluble human CXCL10 was verified to activate murine CXCR3 using a murine T cell line in calcium mobilization assays. Here, murine and human CXCL10 elicited virtually identical signals (Figure S3). The same result was found when murine and human CXCL10 were compared in CXCR3 internalization assays using murine T cells (Figure S3). Subsequently, the GPI-anchored CXCL10 fusion proteins were assessed regarding their ability to induce adhesion of primary murine NK cells under flow. Primary human microvascular endothelial cells were treated with CXCL10-GPI or CXCL10-mucin-GPI or commercially available human CXCL10 at a much higher concentration. Murine NK cells were then perfused over the endothelial cells at a shear rate of 0.4 (Figure 5A) or 1 dyn/cm^2^ (Figure 5B). At the subphysiologic flow rate of 0.4 dyn/cm^2^, 70 events of rolling adhesion and 37 tightly adhering NK cells/mm^2^ were observed for the endothelial cells treated with CXCL10-mucin-GPI. For the CXCL10-GPI protein, 14 rolling and 18 tight adhesions/mm^2^ were seen, suggesting that some adhesion was induced by the membrane-anchored CXCL10 also in the absence of the mucin domain. While few adhesion events (up to 8 events/mm^2^) were seen with soluble CXCL10, this was also true for the medium control. This background adherence was completely abrogated when the physiologic shear rate of 1 dyn/cm^2^ was employed (panel B). Here also the difference between the two CXCL10 fusion proteins was more pronounced. Eight times more cells were tightly bound by the protein containing the mucin domain than by the one lacking it. The difference in the induction of rolling adhesion was also more pronounced at the more physiologic shear rate: No events were observed in the sample with CXCL10-GPI, while CXCL10-mucin-GPI was able to induce 30 rolling adhesions/mm^2^. These results suggested that CXCL10-mucin-GPI is effective also on murine CXCR3 and can recruit murine NK cells within the same range of efficiency as seen with human NK cells. Moreover, the results underlined the importance of the mucin domain for recruitment at physiologic shear stress.

### Activity of the CXCL10-based Fusion Proteins in Established Murine Tumors

**Figure 6:**
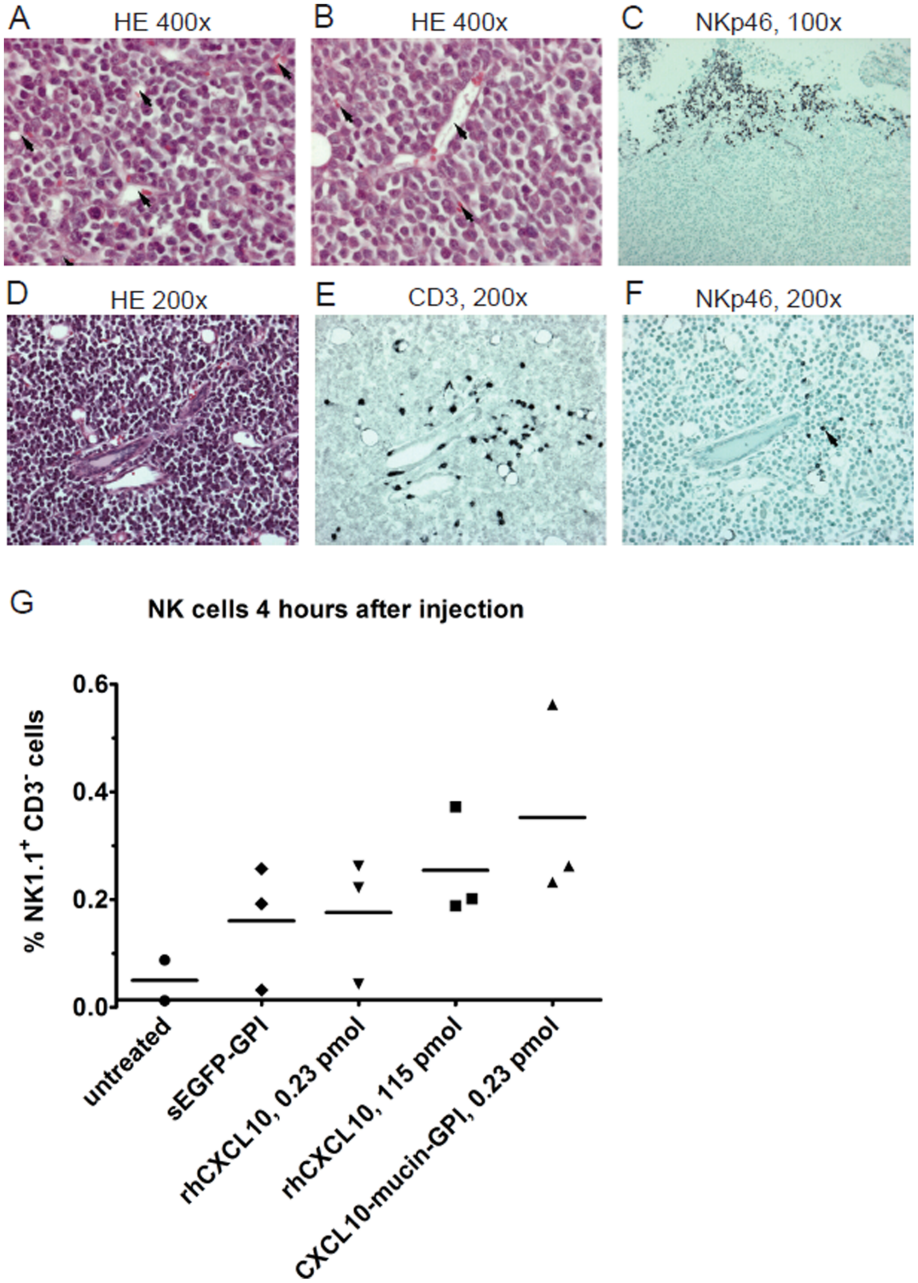
Subcutaneously implanted 291 tumors are well vascularized and show a pronounced infiltration with T cells. Tumor cells were injected subcutaneously into the flanks of C57BL/6 mice. Grown tumors were excised, fixed and analyzed by hematoxylin/eosin (H/E) staining or immunohistology using antibodies against CD3 or NKp46. **A–F**: The respective magnifications are indicated at the top of each image. Panels A and B show the presence of numerous blood vessels (arrows) throughout the tumors in H/E staining. Panels D, E and F represent serial sections of the same position within a tumor. Various blood vessels are visible in H/E staining and the CD3 staining shows infiltration of the tumor with CD3^+^ cells (dark grey/black staining). Less NKp46^+^ cells could be detected in the respective staining of the same position (dark grey/black staining; one cell is marked by an arrow). Panel C depicts a cluster of NKp46^+^ cells, which was sometimes found at the margins of tumors (margin: upper part of the picture). **G**: Injection of CXCL10-mucin-GPI tends to increase endogenous NK cell infiltration of subcutaneous tumors. Purified CXCL10-mucin-GPI was injected into established 291 tumors. As controls, either the same or a 500×higher molar quantity of commercially available human CXCL10 (rhCXCL10) were injected. As additional controls, the same volume of identically purified sEGFP-GPI was injected or the tumors were left completely untreated. The animals were sacrificed 4 h after injection and infiltration of the tumors was assessed by FACS analysis. The figure shows the percentage of CD3^-^ NK1.1^+^ cells among the total lymphocyte count with each symbol representing one individual tumor and horizontal bars the average values. Statistical significance was calculated using the Kruskal-Wallis-test (P = 0.19) followed by Dunńs post test (not significant, N.S.).

A model based on subcutaneously transplantable B cell tumors was used to study the effects of CXCL10-mucin-GPI after intratumoral injection *in vivo* (Figure 6). The 291 B cell lymphoma line [26] was established from a transgenic C57BL/6 mouse carrying the c-myc oncogene under the control of the Igλ promoter [35]. The tumor cells were negative for CXCR3 by FACS and reverse transcriptase real-time PCR (data not shown). Tumor cells were injected into the flanks of wildtype C57BL/6 mice and palpation was used to assess tumor growth. For characterization of tumor tissue, tumors were excised and subjected to hematoxylin/eosin staining (H/E) or immunohistology for CD3 and NKp46 (Figure 6).

The tumors were well vascularized with a high number of blood vessels visible in H/E staining as depicted in panels A and B, and displayed little signs of necrosis. Panels D, E and F represent serial sections of a position near the tumor center. CD3 staining showed pronounced infiltration of the tumor mass with T cells, especially in the vicinity of the blood vessels. NKp46 staining of the same position revealed a much lower infiltration with NK cells compared to CD3^+^ cells. This finding was true for all untreated tumors. At the tumor margins, however, occasionally clusters of NKp46^+^ cells could be found, as exemplified in panel C. These clusters contained only few CD3^+^ cells (data not shown). In summary, CD3^+^ cells were found abundantly and relatively equally distributed throughout the tumors, while NKp46^+^ cells sometimes displayed a highly unequal distribution. For this reason, FACS analysis was used to establish the level of NK cell infiltrate within the tumor tissue in order to avoid skewing of data by image acquisition differences. FACS analysis for T cells was in contrast less informative due to the presence of lymphoid organs removed together with the tumors and high numbers of lymphoid-derived background T cells (data not shown).

In order to assess the effect of the recombinant CXCL10 fusion proteins on lymphocyte recruitment, the purified proteins were injected into the center of each tumor and immune infiltrates were analyzed 4 h later. In immunohistology, the presence of T cells was largely unchanged (high) throughout the tumors in all treatment conditions (data not shown). FACS analysis revealed the presence of NK cells in the tumors at ratios ranging from 0% to 0.55% among the total lymphocytes (CD45^+^ cells) (Figure 6G). Tumors treated with CXCL10-mucin-GPI showed the highest numbers of NK cells (0.34%) compared to tumors that had received any of the other treatments. Tumors injected with the same molar amount of commercially available CXCL10 contained on average only half as many NK cells (0.16%) as CXCL10-mucin-GPI treated tumors. Also 500 x higher molar amounts of commercially available CXCL10 were on average less efficient (0.24%) than CXCL10-mucin-GPI. Differences were not statistically significant due to the small sample size in this experiment. Nevertheless, the data suggested a positive effect of CXCL10-mucin-GPI on the recruitment of NK cells into established tumors *in vivo*, complementing the results obtained *in vitro*.

## Discussion

Chemokines help direct the migration of cells. Because cellular migration plays a central role in many human diseases, numerous therapeutic tools to modify chemokine signaling networks have been evaluated, mostly with the aim of suppressing the recruitment of select cell types [36]. In contrast, in the present study an approach to modify tissue micromilieus was developed that allows the selective recruitment of cells carrying specific chemokine receptors. The approach was based on fusion proteins consisting of an N-terminal chemokine head, linked to the mucin-like domain taken from CX3CL1, with a C-terminal GPI anchor replacing the CX3CL1 transmembrane domain.

This novel class of reagents has diverse potential applications ranging from regenerative medicine, e.g. in the context of myocardial infarction where endothelial progenitor cells need to be recruited, to cancer therapy, where tumor infiltration with anti-neoplastic effector cells needs to be enhanced.

In the present study, examples of fusion proteins were designed and tested in order to investigate the feasibility of this approach. The proteins were designed for application in cancer therapy to facilitate the recruitment of endogenous or adoptively transferred lymphocytes. Our initial focus was on the facilitated recruitment of NK cells.

NK cell infiltration has been identified as a positive prognostic value in various tumors [2], [3], [37]. Poor NK cell infiltration in some tumors has been associated with impaired immune-based tumor control. A requirement for NK/T cell cooperation for full efficacy in lymphocyte control of tumor growth has also been suggested [38]. Thus, the recruitment of effector NK cells represents an important step for effective immunological treatment of tumors.

The adoptive transfer of immune effector cells (T cells or NK cells) has shown therapeutic efficacy in some settings and holds promise for the development of future cancer therapies [39], [40]. As is seen with endogenous antitumor effector cells, one of the major hurdles for a broader application of this treatment modality lies in insufficient infiltration of the tumors by the adoptively transferred cells [7]–[10], [41].

The basis for the defective infiltration of tumors by endogenous or adoptively transferred lymphocytes has been attributed to a reduced interaction of leukocytes with the blood vessel wall [6]–[8], [14]. This phenomenon has been termed endothelial cell anergy and is characterized by reduced expression of intercellular adhesion molecule 1 and 2 (ICAM-1 and -2) and vascular endothelial cell adhesion molecule 1 (VCAM-1) as well as changes in the proteoglycans needed for appropriate presentation of chemokines at the luminal side of endothelial cells [12]–[14]. Without these molecules leukocytes cannot undergo tight adhesion or diapedesis [42], [43]. We sought to overcome this problem by generating a novel, flexible class of reagents that could be used for the targeted modification of tissue micromilieus. According to our hypothesis, recombinant fusion proteins such as CXCL10-mucin-GPI when injected into a solid tumor, incorporate into the cell membranes of tumor, stromal and endothelial cells [19]. When present on tumor endothelial cells, the proteins should help overcome endothelial cell anergy by specifically recruiting leukocytes that express the matching chemokine receptor with limited requirement for other adhesion molecules.

We show here that CXCL10-mucin-GPI is bioactive as it is able to bind and activate the CXCL10 receptor CXCR3. Purified proteins incorporated readily into the cell membranes of CHO cells as well as microvascular endothelial cells. In our *in vitro* assay system, the protein was able to recruit freely flowing NK cells under conditions of physiologic flow following incorporation into primary microvascular endothelial cells. This process was shown to be dependent on the presence of the mucin domain. The results obtained in a mouse tumor model documenting a trend towards an enhanced NK cell recruitment into CXCL10-mucin-GPI treated lymphoma nodules suggested that the positive effects hold true also *in vivo* in a tumor setting. Following this first proof of principle, the underlying concept of a chemokine head fused to the mucin domain of CX3CL1 and a GPI anchor signal sequence may be expanded into a broader family of reagents that allow targeted recruitment of cells into various tissues. Along this line, we currently have a manuscript in press describing the use of a similar reagent which contains a CXCL12 (SDF-1) head in the recruitment of CXCR4^+^ stem cells [44]. When expressed on endothelial cells *in vitro*, this reagent effectively induced adhesion of CXCR4^+^ cells. Moreover, if expressed *in situ*, it enhanced the recruitment of endothelial progenitor cells in a rabbit hindlimb ischemia model *in vivo*, leading to an enhanced perfusion post ischemia.

## Supporting Information

Figure S1 **Human CXCL10 induces CXCR3 internalization and Calcium flux in murine T cells.** In order to confirm cross-reactivity of the human-based CXCL10 fusion proteins in the murine system, receptor internalization and Calcium mobilization assays were performed. These complement the flow assay experiments presented in figure 5 of the main manuscript. Murine CD4^+^ CXCR3^+^ T cells (kindly provided by Frank Lehmann, Helmholtz-Zentrum Munich) were used as target cells in both assays. Murine CXCL10 was obtained from Peprotech, Rocky Hill. **A**: Calcium mobilization. Murine T cells were loaded with Fluo-4 according to the manufactureŕs instructions. Flow cytometry was used to measure the fluorescence in the cell population. 30 seconds after the beginning of the assay (indicated by the black arrow), 1 µg/ml human (orange line) or murine (blue line) CXCL10 or buffer (black line) was added and the fluorescence was measured for another 3 minutes. Human and murine CXCL10 induced virtually identical Calcium responses in the T cells. **B**: Receptor internalization. The assay was performed essentially as described in the main manuscript, but using murine T cells instead of human cells. The cells were incubated with 1 µg/ml human or murine CXCL10 or a buffer control for 30 min and subsequently stained with CXCR3-specific antibodies and analyzed by flow cytometry. The figure shows relative fluorescence levels normalized to the ones found in the buffer-treated control cells. Both human and murine CXCL10 induced similar levels of CXCR3 internalization, indicative of cross-reactivity of the human protein in murine cells.
(TIF)

Figure S2 **CXCR3 internalization by the CXCL10 fusion proteins.** As shown in figure 2, panel C of the main manuscript, the CXCL10 fusion proteins induced internalization of CXCR3 on cells of a human T cell line. To exclude the possibility that the decrease in signal intensity was due to occupation of CXCR3 by CXCL10 leading to decreased accessibility of the epitope for the detection antibody instead of an actual internalization of the receptor, additional experiments were performed. First, the coincubation was performed at 4°C instead of 37°C in order to slow down cellular activity. This lead to a much attenuated CXCR3 internalization, consistent with an actual receptor internalization, which is an active process that is slowed with decreasing temperature. Second, a different antibody clone was used for the detection of CXCR3, which yielded similar results as the antibody clone used in the experiments presented in the main manuscript.
(TIF)

Figure S3 **Calcium mobilization by the CXCL10 fusion proteins.** A transient rise in the cytoplasmic calcium concentration is frequently used to monitor chemokine receptor activation and the initiation of downstream signaling [2]. Coincubation experiments were performed to assess the ability of the recombinant CXCL10 fusion proteins to induce Calcium mobilization in CXCR3^+^ cells. Human T cells (JB4) were loaded with Fluo-4 according to the manufactureŕs instructions, centrifuged and resuspended in fresh assay buffer to yield 5×10^6^ cells/ ml. 50 µl of this suspension were transferred into each well of a 96 well flat bottom plate. The same number of wells was filled with 50 µl of assay buffer only as control. Subsequently, 50 µl of non-transfected CHO cells or cells transfected with the recombinant CXCL10 fusion proteins suspended in assay buffer (1×10^7^ cells/ ml) were added simultaneously to wells containing labeled DS4 cells or assay buffer. Measurements were performed in a microlate-reader (485 nm excitation wavelength and 535 nm emission wavelength) every 20 sec over a period of 40 min, during which the plate was kept heated to 37°C. All samples were run in duplicates. To compensate for CHO cell autofluorescence, readings that had been taken in the samples in which the respective CHO cells had been “coincubated” with assay buffer only were subtracted for each time point from the readings that had been taken in samples in which the respective CHO cells had been coincubated with DS4 cells. For better readability, readings are shown as Δ fluorescence values against coincubation with non-transfected CHO. Commercially available soluble CXCL10 was used in separate wells as positive control for the loading procedure and the function of the Fluo-4 dye (data not shown). Before the experiment, the transfected CHO cells were assayed for the expression levels of the recombinant fusion proteins by FACS analysis and found to express the proteins at similar levels (data not shown). During the coincubation time of 40 min, a transient increase of the fluorescence intensities could be observed, indicating Calcium mobilization in the T cells. The relatively long period of 40 min, over the course of which the increased fluorescence was observed, may have resulted from single contacts between T cells and CHO cells - each resulting in short-lived calcium mobilizations - until eventually all T cells had been desensitized for CXCL10. Interestingly, CHO cells transfected with CXCL10-mucin-GPI induced a much faster and stronger calcium response than cells transfected with CXCL10-GPI. It is possible that the mucin domain facilitated the interaction of the chemokine head with CXCR3 on T cells by presenting the chemokine domain away from the cell surface.
(TIF)

Materials and Methods S1 **Supplemental Materials and Methods.**
(DOCX)

Video S1 **Endothelium treated with recombinant CXCL10 and endothelium treated with CXCL10-mucin-GPI.** The videos show representative experiments, in which YT cells were perfused over an endothelial cell monolayer treated with recombinant CXCL10 or CXCL10-mucin-GPI at 1 dyn/cm^2^ as detailed in the main manuscript (compare figure 4). The videos are shown in real-time. After 10 or 20 seconds, the videos jump to the end of the 5-minute observation period in each experiment. No adhesions can be seen on the endothelial cells treated only with recombinant CXCL10, while an accumulation of NK cells is evident on endothelial cells treated with the CXCL10-mucin-GPI fusion protein.
(Mp4)

Video S2 **Rolling and tight adhesion.** The video shows a small section of endothelium treated with CXCL10-mucin-GPI as detailed in the main manuscript in 16x fast motion. YT cells are perfused over the endothelial cells at 1 dyn/cm^2^, The three cells that are captured by the endothelial cells in the first half of the video eventually reach tight adhesion, whereas several events of rolling adhesion can be seen in the second half of the video.
(Mp4)
