# Supplementary figures and images for: A Novel CXCL10-Based GPI-Anchored Fusion Protein as Adjuvant in NK-Based Tumor Therapy
Source: PLoS One. 2013 Aug 30;8(8):e72749. doi: 10.1371/journal.pone.0072749 (PMC3758322; doi:10.1371/journal.pone.0072749)

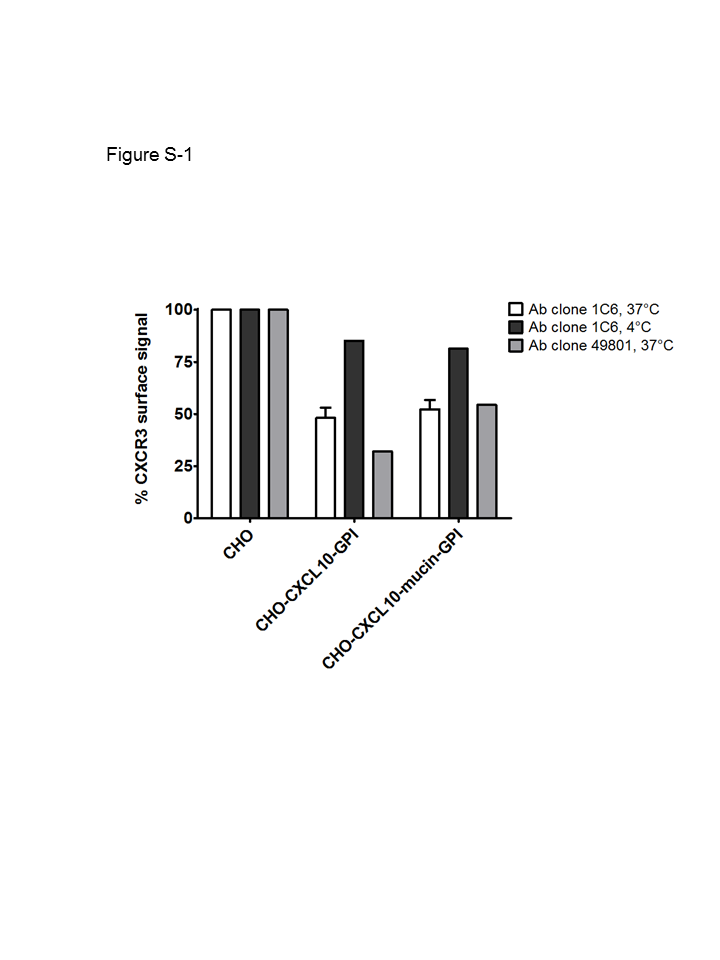

Supplement: Figure S1 — Human CXCL10 induces CXCR3 internalization and Calcium flux in murine T cells. In order to confirm cross-reactivity of the human-based CXCL10 fusion proteins in the murine system, receptor internalization and Calcium mobilization assays were performed. These complement the flow assay experiments presented in figure 5 of the main manuscript. Murine CD4+ CXCR3+ T cells (kindly provided by Frank Lehmann, Helmholtz-Zentrum Munich) were used as target cells in both assays. Murine CXCL10 was obtained from Peprotech, Rocky Hill. A: Calcium mobilization. Murine T cells were loaded with Fluo-4 according to the manufactureŕs instructions. Flow cytometry was used to measure the fluorescence in the cell population. 30 seconds after the beginning of the assay (indicated by the black arrow), 1 µg/ml human (orange line) or murine (blue line) CXCL10 or buffer (black line) was added and the fluorescence was measured for another 3 minutes. Human and murine CXCL10 induced virtually identical Calcium responses in the T cells. B: Receptor internalization. The assay was performed essentially as described in the main manuscript, but using murine T cells instead of human cells. The cells were incubated with 1 µg/ml human or murine CXCL10 or a buffer control for 30 min and subsequently stained with CXCR3-specific antibodies and analyzed by flow cytometry. The figure shows relative fluorescence levels normalized to the ones found in the buffer-treated control cells. Both human and murine CXCL10 induced similar levels of CXCR3 internalization, indicative of cross-reactivity of the human protein in murine cells. (TIF) [file pone.0072749.s001.tif]

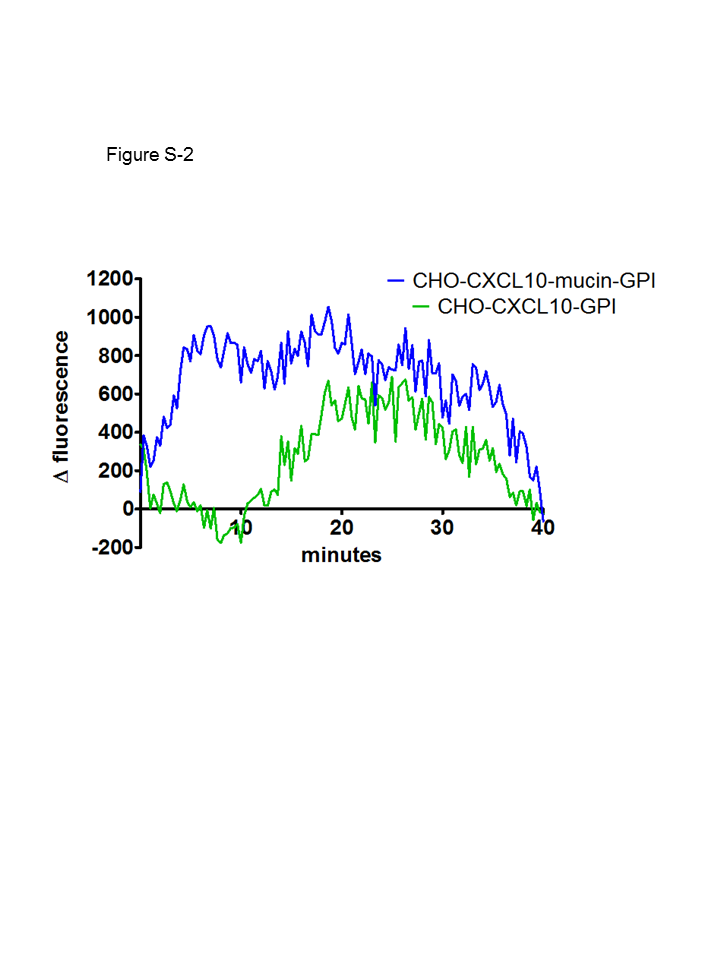

Supplement: Figure S2 — CXCR3 internalization by the CXCL10 fusion proteins. As shown in figure 2, panel C of the main manuscript, the CXCL10 fusion proteins induced internalization of CXCR3 on cells of a human T cell line. To exclude the possibility that the decrease in signal intensity was due to occupation of CXCR3 by CXCL10 leading to decreased accessibility of the epitope for the detection antibody instead of an actual internalization of the receptor, additional experiments were performed. First, the coincubation was performed at 4°C instead of 37°C in order to slow down cellular activity. This lead to a much attenuated CXCR3 internalization, consistent with an actual receptor internalization, which is an active process that is slowed with decreasing temperature. Second, a different antibody clone was used for the detection of CXCR3, which yielded similar results as the antibody clone used in the experiments presented in the main manuscript. (TIF) [file pone.0072749.s002.tif]

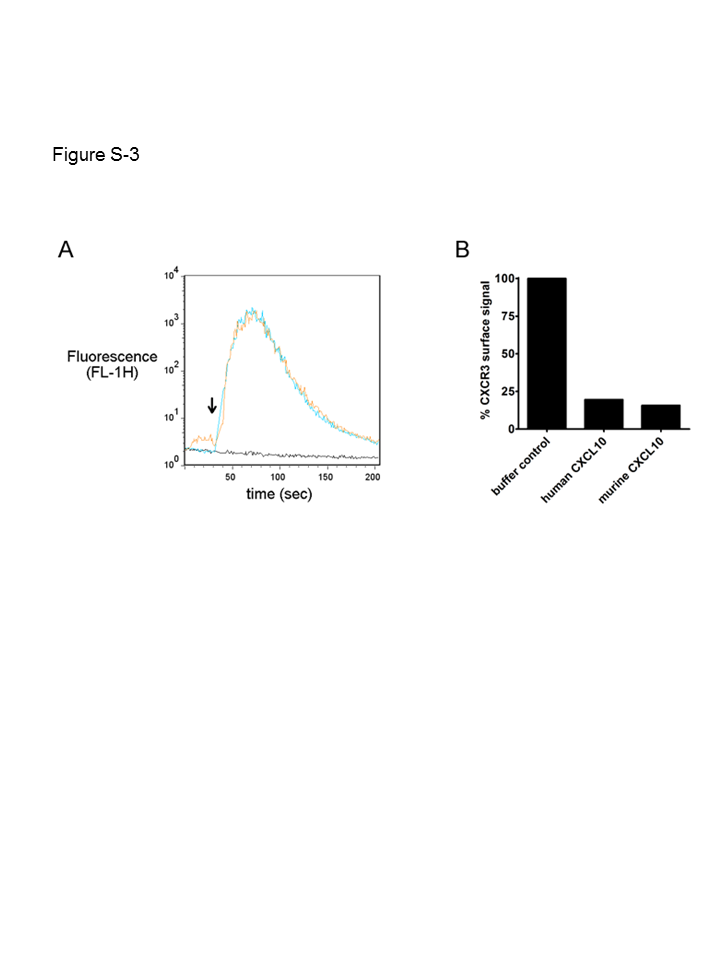

Supplement: Figure S3 — Calcium mobilization by the CXCL10 fusion proteins. A transient rise in the cytoplasmic calcium concentration is frequently used to monitor chemokine receptor activation and the initiation of downstream signaling [2]. Coincubation experiments were performed to assess the ability of the recombinant CXCL10 fusion proteins to induce Calcium mobilization in CXCR3+ cells. Human T cells (JB4) were loaded with Fluo-4 according to the manufactureŕs instructions, centrifuged and resuspended in fresh assay buffer to yield 5×10^6^ cells/ ml. 50 µl of this suspension were transferred into each well of a 96 well flat bottom plate. The same number of wells was filled with 50 µl of assay buffer only as control. Subsequently, 50 µl of non-transfected CHO cells or cells transfected with the recombinant CXCL10 fusion proteins suspended in assay buffer (1×10^7^ cells/ ml) were added simultaneously to wells containing labeled DS4 cells or assay buffer. Measurements were performed in a microlate-reader (485 nm excitation wavelength and 535 nm emission wavelength) every 20 sec over a period of 40 min, during which the plate was kept heated to 37°C. All samples were run in duplicates. To compensate for CHO cell autofluorescence, readings that had been taken in the samples in which the respective CHO cells had been “coincubated” with assay buffer only were subtracted for each time point from the readings that had been taken in samples in which the respective CHO cells had been coincubated with DS4 cells. For better readability, readings are shown as Δ fluorescence values against coincubation with non-transfected CHO. Commercially available soluble CXCL10 was used in separate wells as positive control for the loading procedure and the function of the Fluo-4 dye (data not shown). Before the experiment, the transfected CHO cells were assayed for the expression levels of the recombinant fusion proteins by FACS analysis and found to express the proteins at similar levels (data not shown). During th [file pone.0072749.s003.tif]
