# Supplementary material for: A Novel CXCL10-Based GPI-Anchored Fusion Protein as Adjuvant in NK-Based Tumor Therapy
Source: PLoS One. 2013 Aug 30;8(8):e72749. doi: 10.1371/journal.pone.0072749 (PMC3758322; doi:10.1371/journal.pone.0072749)
Supplement: Materials and Methods S1 — Supplemental Materials and Methods. (DOCX) [file pone.0072749.s004.docx]

# Supplemental materials

**Materials and Methods**

## Molecular cloning strategies:

For detection and purification purposes all recombinant constructs were equipped with a c-myc epitope tag directly 5´ of the GPI anchor signal sequence if present, otherwise at the 3´ end of the respective construct.

*CXCL10-GPI:* The GPI anchor signal sequence derived from LFA-3 (amino acids 203-232 of GenBank entry NM_001799.2) was amplified as described [[1](#_ENREF_1)]. A c-myc epitope tag was amplified from the retroviral vector MP71 (kind gift from Dr. Uckert, Berlin, Germany; primers: see below) and ligated into the plasmid, 5´ of the LFA3 GPI anchor signal sequence. The CXCL10 gene was PCR-amplified without stop codon (primers: see supplemental materials) and ligated into the vector. The construct was then subcloned into a pEF_dhfr_ vector (kind gift of M. Mack, Regensburg, Germany) for expression in CHO^dhfr-/-^ cells. Selection of transfected cells was performed using the dihydrofolatereductase gene in the pEF_dhfr_ vector as selection marker and dialyzed serum devoid of nucleotides to generate selective pressure.

*CXCL10-mucin-GPI:* The mucin domain of CX3CL1 (amino acids 100-341 of GeneBank entry BC016164.1) was amplified from a human inflamed kidney cDNA sample (primers: see below) and inserted between the CXCL10 gene sequence and the c-myc epitope tag.

*CXCL10-mucin-Stop:* The c-myc tag and the GPI anchor signal sequence was removed from the CXCL10 -GPI plasmid and replaced by a cassette consisting of the CX3CL1 mucin domain gene and the c-myc tag sequence followed by a stop codon. The resulting CXCL10-mucin-Stop gene was then subcloned into a pEF_dhfr_ vector.

*sEGFP-GPI:* An N-terminal (5´) secretion signal sequence and the C-terminal (3´) GPI anchor signal sequence was added to the EGFP gene. The secretion signal sequence was taken from human tissue inhibitor of matrix metalloproteases 1 (TIMP-1; amino acids 1-23 of GeneBank entry NM_003254.2). A double c-myc tag was included between the eGFP gene and the GPI signal sequence. The resulting construct termed sEGFP-GPI (s for secretion signal sequence) was subcloned into pEF_dhfr_.

## Protein purification

The GPI-anchored fusion proteins were purified using the double c‑myc epitope tag integrated into all constructs in combination with an affinity resin as described by the manufacturer (MBL, Woburn, USA). 1.5 x 10^8^ transfected CHO cells were harvested using EDTA and stored at -80°C until use. For protein purification, cells were resuspended in 10 ml extraction buffer (4 mM n-Dodecyl-β-D-Maltoside (Calbiochem/Merck, Darmstadt, Germany); 50 mMTris/HCl, pH 7.5; 100 mMNaCl; protease inhibitors (*complete* tablets, Roche, Mannheim)). Cell suspensions were rotated for 1 h at 4°C and centrifuged at 16,000 x g for 20 min (4°C) to remove cell debris. The supernatant was sterile filtered prior to chromatography. The respective c-myc affinity column was equilibrated in PBS + 0.025% hydrogenated Triton X-100 (Calbiochem/Merck, Darmstadt) and the cell extract was applied to the column with 0.01 ml/min flow rate. In order to elute non-specifically bound proteins, 3 CV of high-salt washing buffer (PBS + 145 mM NaCl) were perfused through the column, followed by equilibration buffer, and bound proteins were eluted by injecting 5 ml of equilibration buffer containing 0.01 mg/ml c-myc peptide.

All fractions were assayed for their content of the respective fusion proteins by western blotting using anti-c-myc antibodies (clone 9E10, purified in house) and reducing conditions. In the case of the sEGFP fusion proteins, the fluorescence (485 nm excitation, 535 nm emission) was measured instead of western blotting. Elution fractions containing the highest amount of fusion protein were pooled and the elution pool was concentrated using ultrafiltration devices with a molecular size cutoff of 5 kDa. The specific concentrations of the chemokine fusion proteins were determined using a commercially available CXCL10 ELISA kit (R&D Systems, Minneapolis, USA) according to the manufacturer´s instructions. Protein purity was assessed using SDS-PAGE and silver staining as well as a comparison between the specific protein content (ELISA) and the total protein content (BCA Assay). Purified proteins were stored at 4°C and used for up to 1 week.

## Fluorescence activated cell scanning (FACS) analyses of expressed fusion proteins

Cells were detached using ETDA, washed and resuspended in FACS buffer (1x10^6^ cells per sample) containing monoclonal antibodies (anti c-myc: clone 9E10, purified in house, 10 µg/ml; anti CXCL10: BD Biosciences, Bedford, USA, 10 µg/ml; anti CX3CL1 mucin domain: Abnova, Taipei, Taiwan, 5 µg/ml), or isotype-matched control antibodies (Sigma-Aldrich, Taufkirchen, Germany). Following incubation for 45 min at 4°C, the cells were washed and resuspended in FACS buffer containing RPE- or FITC-labeled secondary antibodies (Dako, Roskilde, Denmark, 10 µg/ml) and 4 µg/ml 7-AAD. After 30 min incubation at 4°C, the cells were washed again and analyzed.

**Calcium mobilization in T cells**

Coincubation experiments with T cells and CHO cells were performed to assess the ability of the recombinant CXCL10 fusion proteins to induce calcium mobilization in CXCR3^+^ cells. Human T cells (DS4) were loaded with Fluo-4 (Invitrogen, Carlsbad, USA) according to the manufacturer´s instructions, centrifuged and resuspended in fresh assay buffer to yield 5 x 10^6^ cells/ml. 50 µl of this suspension were transferred into each well of a 96 well flat bottom plate. The same number of wells was filled with 50 µl of assay buffer only as control. Subsequently, 50 µl of non-transfected CHO cells or CHO cells transfected with CXCL10-GPI or CXCL10-mucin-GPI suspended in assay buffer (1 x 10^7^ cells/ml) were added simultaneously to wells containing labeled DS4 cells or assay buffer. Measurements were performed in a microplate-reader (485 nm excitation wavelength and 535 nm emission wavelength) every 20 sec over a period of 40 min, during which the plate was kept heated to 37°C. All samples were run in duplicates. To compensate for CHO cell autofluorescence, readings that had been taken in the samples in which the respective CHO cells had been “incubated” with assay buffer only, were subtracted for each time point from the readings that had been taken in samples in which the respective CHO cells had been coincubated with DS4 cells. The graphical presentation shows the Δ fluorescence values against coincubation with non-transfected CHO.

**Histology of tumor sections**

Fixed tumors were dehydrated with an automatic tissue-processor (Thermo Fisher, Waltham, USA). Paraffin blocks were prepared using liquid paraffin. After cooling, 2 µm sections were cut from these blocks. Endogenous peroxidase activity was blocked by incubating the slides in 3% hydrogen peroxide in methanol for 20 min in the dark. Antigen retrieval was performed using antigen unmasking solution (Vector laboratories, Burlingame, USA) in an autoclave oven for 20 min (CD3 staining) or 50 µg/ml proteinase K for 10 min at room temperature (NKp46 staining). Endogenous Biotin was blocked using a commercially available Avidin/Biotin blocking kit (Vector laboratories, Burlingame, USA). Subsequently, the slides were incubated with CD3-specific antibodies (AbD Serotec, Kidlington, UK; 10 µg/ml, diluted in PBS, for 1 h at rt), NKp46-specific antibodies (R&D Systems, Minneapolis, USA; 10 µg/ml, diluted in 10% skimmed milk powder in PBS, for 1 h at rt) or respective controls. Following incubation with biotinylated secondary antibodies (Vector laboratories, Burlingame, USA; 5 µg/ml in PBS) for 30 min, a commercially available kit was used to detect bound antibodies (*Vectastain*, Vector laboratories, Burlingame, USA) according to the manufacturer´s instructions. 3,3´-Diaminobenzidine (3 mM) was used as substrate diluted in Tris/HCl, pH 7.7 in combination with NiCl_2_ (1.7 mM) and H_2_O_2_ (0.075 ‰). Slides were counter stained using methyl green.

H/E staining was performed to assess the general morphology of the tumor tissue. Following deparaffinisation, the slides were washed using distilled water, stained for 5 min in Harris modified hematoxylin solution (Sigma Aldrich, Taufkirchen, Germany), washed for 5 min in tab water for bluing, incubated in 70% ethanol for 2 min and in eosin Y solution (Sigma Aldrich, Taufkirchen, Germany) for 30 sec. Subsequently, the slides were washed once in 70%, twice in 96% and 3 times in 100% ethanol, followed by xylol.

## Primer sequences

| **Application** | **Primer name** | **Sequence** |
| --- | --- | --- |
| Amplification of c-myc tag | p2xMycTag_fw | 5´- GTTAAGCTGTGTATCTAGAGAACAGAA-3´ |
|  | p2xMycTag_rv | 5´- CTTCATTGCTAGCCAGGTCCTCCTC-3´ |
| Amplification of CX3CL1 mucin domain | Fra_fw_080901 | 5´-GAGAATTCATCTAGAAATGGCGGCACCTTCG-3´ |
|  | Fra_rv_080901 | 5´-GGATACAGGTTGTGCTAGCCTGCCTC-3´ |
| Amplification of CXCL10 | IP10_fw_long | 5´-GAGGAACCTGAATTCCCAGTCTCAGCACC-3´ |
|  | IP10_rv_long | 5´-CCCCTCTGGTGCTAGCAGGAGATCTTTTAG |
| Amplification of EGFP from pEGFP-N1 | EGFP_fw_0408 | 5´-GATCCACCGACGCGTGCCATGGTGAGC-3´ |
|  | EGFP_rv_0408 | 5´-GAGTCGCGGCCTCTAGACTTGTACAGCTCGTCC-3´ |
| Mut. of TIMP-1 signal sequence (*Mlu*I site) | SS_mut_fw | 5´-GGCTGATAGCCCCCACGCGTGCCTGCACCTGTGTC-3´ |
|  | SS_mut_rv | 5´-GACACAGGTGCAGGCACGCGTGGGGGCTATCAGCC-3´ |

## DNA sequences of the recombinant fusion proteins

Start and stop codons in the respective constructs are underlined, available restriction sites are printed in bold.

| **Restriction sites** | **Genes or gene segments** |
| --- | --- |
| **GAATTC**: *Eco*RI | CXCL10 gene |
| **GTCGAC**: *Sal*I | EGFP gene |
| **GCTAGC**: *Nhe*I | Double c-myc epitope tag |
| **TCTAGA**: *Xba*I | Mucin domain from CX3CL1 |
| **ACGCGT**: *Mlu*I | GPI signal sequence from LFA-3 |
|  | Secretion signal sequence from TIMP-1 |

**>CXCL10-GPI**

**GAATTC**CCAGTCTCAGCACCATGAATCAAACTGCCATTCTGATTTGCTGCCTTATCTTTCTGACTCTAAGTGGCATTCAAGGAGTACCTCTCTCTAGAACTGTACGCTGTACCTGCATCAGCATTAGTAATCAACCTGTTAATCCAAGGTCTTTAGAAAAACTTGAAATTATTCCTGCAAGCCAATTTTGTCCACGTGTTGAGATCATTGCTACAATGAAAAAGAAGGGTGAGAAGAGATGTCTGAATCCAGAATCGAAGGCCATCAAGAATTTACTGAAAGCAGTTAGCAAGGAAAGGTCTAAAAGATCTCCTGCTAGAGAACAGAAGCTGATCAGCGAGGAGGACCTGGAGCAGAAGTTGATCAGCGAGGAGGACCTGGCTAGAACAACCTGTATCCCAAGCAGCGGTCATTCAAGACACAGATATGCACTTATACCCATACCATTAGCAGTAATTACAACATGTATTGTGCTGTATATGAATGTATTATGA**GTCGAC**

**>CXCL10-mucin-GPI**

**GAATTC**CCAGTCTCAGCACCATGAATCAAACTGCCATTCTGATTTGCTGCCTTATCTTTCTGACTCTAAGTGGCATTCAAGGAGTACCTCTCTCTAGAACTGTACGCTGTACCTGCATCAGCATTAGTAATCAACCTGTTAATCCAAGGTCTTTAGAAAAACTTGAAATTATTCCTGCAAGCCAATTTTGTCCACGTGTTGAGATCATTGCTACAATGAAAAAGAAGGGTGAGAAGAGATGTCTGAATCCAGAATCGAAGGCCATCAAGAATTTACTGAAAGCAGTTAGCAAGGAAAGGTCTAAAAGATCTCCTGCTAGAAATGGCGGCACCTTCGAGAAGCAGATCGGCGAGGTGAAGCCCAGGACCACCCCTGCCGCCGGGGGAATGGACGAGTCTGTGGTCCTGGAGCCCGAAGCCACAGGCGAAAGCAGTAGCCTGGAGCCGACTCCTTCTTCCCAGGAAGCACAGAGGGCCCTGGGGACCTCCCCAGAGCTGCCGACGGGTGTGACTGGTTCCTCAGGGACCAGGCTCCCCCCGACGCCAAAGGCTCAGGATGGAGGGCCTGTGGGCACGGAGCTTTTCCGAGTGCCTCCCGTCTCCACTGCCGCCACGTGGCAGAGTTCTGCTCCCCACCAACCTGGGCCCAGCCTCTGGGCTGAGGCAAAGACCTCTGAGGCCCCGTCCACCCAGGACCCCTCCACCCAGGCCTCCACTGCGTCCTCCCCAGCCCCAGAGGAGAATGCTCCGTCTGAAGGCCAGCGTGTGTGGGGTCAGGGGCAGAGCCCCAGGCCAGAGAACTCTCTGGAGCGGGAGGAGATGGGTCCCGTGCCAGCGCACACGGATGCCTTCCAGGACTGGGGGCCTGGCAGCATGGCCCACGTCTCTGTGGTCCCTGTCTCCTCAGAAGGGACCCCCAGCAGGGAGCCAGTGGCTTCAGGCAGCTGGACCCCTAAGGCTGAGGAACCCATCCATGCCACCATGGACCCCCAGAGGCTGGGCGTCCTTATCACTCCTGTCCCTGACGCCCAGGCTGCCACCCGGAGGCAGGCTAGAGAACAGAAGCTGATCAGCGAGGAGGACCTGGAGCAGAAGTTGATCAGCGAGGAGGACCTG**GCTAGC**ACAACCTGTATCCCAAGCAGCGGTCATTCAAGACACAGATATGCACTTATACCCATACCATTAGCAGTAATTACAACATGTATTGTGCTGTATATGAATGTATTATGA**GTCGAC**

**>CXCL10-mucin-Stop**

**GAATTC**CCAGTCTCAGCACCATGAATCAAACTGCCATTCTGATTTGCTGCCTTATCTTTCTGACTCTAAGTGGCATTCAAGGAGTACCTCTCTCTAGAACTGTACGCTGTACCTGCATCAGCATTAGTAATCAACCTGTTAATCCAAGGTCTTTAGAAAAACTTGAAATTATTCCTGCAAGCCAATTTTGTCCACGTGTTGAGATCATTGCTACAATGAAAAAGAAGGGTGAGAAGAGATGTCTGAATCCAGAATCGAAGGCCATCAAGAATTTACTGAAAGCAGTTAGCAAGGAAAGGTCTAAAAGATCTCCTGCTAGAAATGGCGGCACCTTCGAGAAGCAGATCGGCGAGGTGAAGCCCAGGACCACCCCTGCCGCCGGGGGAATGGACGAGTCTGTGGTCCTGGAGCCCGAAGCCACAGGCGAAAGCAGTAGCCTGGAGCCGACTCCTTCTTCCCAGGAAGCACAGAGGGCCCTGGGGACCTCCCCAGAGCTGCCGACGGGTGTGACTGGTTCCTCAGGGACCAGGCTCCCCCCGACGCCAAAGGCTCAGGATGGAGGGCCTGTGGGCACGGAGCTTTTCCGAGTGCCTCCCGTCTCCACTGCCGCCACGTGGCAGAGTTCTGCTCCCCACCAACCTGGGCCCAGCCTCTGGGCTGAGGCAAAGACCTCTGAGGCCCCGTCCACCCAGGACCCCTCCACCCAGGCCTCCACTGCGTCCTCCCCAGCCCCAGAGGAGAATGCTCCGTCTGAAGGCCAGCGTGTGTGGGGTCAGGGGCAGAGCCCCAGGCCAGAGAACTCTCTGGAGCGGGAGGAGATGGGTCCCGTGCCAGCGCACACGGATGCCTTCCAGGACTGGGGGCCTGGCAGCATGGCCCACGTCTCTGTGGTCCCTGTCTCCTCAGAAGGGACCCCCAGCAGGGAGCCAGTGGCTTCAGGCAGCTGGACCCCTAAGGCTGAGGAACCCATCCATGCCACCATGGACCCCCAGAGGCTGGGCGTCCTTATCACTCCTGTCCCTGACGCCCAGGCTGCCACCCGGAGGCAGGCTAGAGAACAGAAGCTGATCAGCGAGGAGGACCTGGAGCAGAAGTTGATCAGCGAGGAGGACCTGTA**GTCGAC**

**>sEGFP-GPI**

**GAATTC**ATGGCCCCCTTTGAGCCCCTGGCTTCTGGCATCCTGTTGTTGCTGTGGCTGATAGCCCCC**ACGCGT**GCCATGGTGAGCAAGGGCGAGGAGCTGTTCACCGGGGTGGTGCCCATCCTGGTCGAGCTGGACGGCGACGTAAACGGCCACAAGTTCAGCGTGTCCGGCGAGGGCGAGGGCGATGCCACCTACGGCAAGCTGACCCTGAAGTTCATCTGCACCACCGGCAAGCTGCCCGTGCCCTGGCCCACCCTCGTGACCACCCTGACCTACGGCGTGCAGTGCTTCAGCCGCTACCCCGACCACATGAAGCAGCACGACTTCTTCAAGTCCGCCATGCCCGAAGGCTACGTCCAGGAGCGCACCATCTTCTTCAAGGACGACGGCAACTACAAGACCCGCGCCGAGGTGAAGTTCGAGGGCGACACCCTGGTGAACCGCATCGAGCTGAAGGGCATCGACTTCAAGGAGGACGGCAACATCCTGGGGCACAAGCTGGAGTACAACTACAACAGCCACAACGTCTATATCATGGCCGACAAGCAGAAGAACGGCATCAAGGTGAACTTCAAGATCCGCCACAACATCGAGGACGGCAGCGTGCAGCTCGCCGACCACTACCAGCAGAACACCCCCATCGGCGACGGCCCCGTGCTGCTGCCCGACAACCACTACCTGAGCACCCAGTCCGCCCTGAGCAAAGACCCCAACGAGAAGCGCGATCACATGGTCCTGCTGGAGTTCGTGACCGCCGCCGGGATCACTCTCGGCATGGACGAGCTGTACAAG**TCTAGA**GAACAGAAGCTGATCAGCGAGGAGGACCTGGAGCAGAAGTTGATCAGCGAGGAGGACCTG**GCTAGC**ACAACCTGTATCCCAAGCAGCGGTCATTCAAGACACAGATATGCACTTATACCCATACCATTAGCAGTAATTACAACATGTATTGTGCTGTATATGAATGTATTATGA**GTCGAC**

1. Notohamiprodjo M, Djafarzadeh R, Mojaat A, von Luttichau I, Grone HJ, et al. (2006) Generation of GPI-linked CCL5 based chemokine receptor antagonists for the suppression of acute vascular damage during allograft transplantation. Protein Eng Des Sel 19: 27-35.
